# NCAPH as a potential prognostic signaling biomarker regulating low-grade glioma cell proliferation, migration, invasion, immune microenvironment, and drug sensitivity

**DOI:** 10.7150/jca.115870

**Published:** 2025-09-27

**Authors:** Lirui Dai, Shu Jiang, Peizhi Zhou

**Affiliations:** Department of Neurosurgery, West China Hospital of Sichuan University, Sichuan University, Chengdu, Sichuan, China.

**Keywords:** NCAPH, low-grade glioma, prognosis biomarker, tumor immune microenvironment

## Abstract

NCAPH is mainly involved in the transformation of nuclear chromatin in the intercellular phase into a highly heliform nuclear chromosome, encoded by a gene on chromosome 2q11.2. Studies have found that NCAPH, as an oncogene, plays important roles in the occurrence and development of several cancers, significantly affecting the survival and prognosis of patients. However, the role of NCAPH in low-grade glioma (LGG) has been largely unexplored. Here, we conduct a comprehensive analysis of NCAPH expression, abundance of cell subsets in single-cell cohorts, prognosis, co-localization, epigenetic alterations, functional enrichment, tumor immune-related features, immunotherapy response, drug sensitivity, and molecular docking in LGG. Our findings suggest that NCAPH is significantly overexpressed in LGG and is strongly relevant to poor prognosis, and that NCAPH plays important roles in reshaping the tumor microenvironment, which may promote immune tolerance of LGG and thus become a potential immunotherapeutic target. The sensitivity of LGG patients with high NCAPH expression to chemotherapy agents such as temozolomide also suggests the potential therapeutic effect of chemotherapy combined with immunotherapy. Furthermore, NCAPH was positively correlated with cell cycle, proliferation, DNA damage and repair, EMT, invasion, and apoptosis, while negatively associated with inflammation, quiescence and angiogenesis. Together, this study provides a comprehensive understanding of the role of NCAPH in LGG and suggests that NCAPH may be a potential prognostic biomarker for LGG patients, with potential for drug development and immunotherapy.

## 1. Introduction

Glioma, representing 25.5% of all primary central nervous system tumors with an annual incidence of 3.38 per 100,000 population 1, presents a formidable challenge in neuro-oncology. Despite multimodal therapeutic approaches including maximal safe resection, radiotherapy, and temozolomide-based chemotherapy, the prognosis remains poor with median survival for glioblastoma patients barely exceeding 15 months 2. This therapeutic stagnation stems from three fundamental challenges: (1) the blood-brain barrier's restriction on drug delivery 3, (2) profound inter- and intra-tumoral heterogeneity 4, and (3) the dynamic evolution of treatment resistance mechanisms 5. Recent advances in nanomedicine have demonstrated that engineered exosomes and surface-modified nanoparticles can overcome biological barriers to achieve targeted drug delivery 6, while emerging immunotherapies have shown limited but promising results in subset populations 7.

The tumor immune microenvironment (TIME) of gliomas exhibits unique immunosuppressive characteristics distinct from peripheral tumors, featuring predominant myeloid cell infiltration, low T-cell prevalence, and particular immune checkpoint expression patterns 8. While immune checkpoint inhibitors have revolutionized treatment in many malignancies, their efficacy in glioma remains constrained by blood-brain barrier penetration issues and intrinsic resistance mechanisms 9. Recent studies reveal that glioma cells actively remodel their microenvironment through exosome-mediated intercellular communication 10, 11, suggesting potential novel immunomodulatory targets, so there is an urgent requirement to discover potential therapeutic targets to effectively enhance the overall survival rate of glioma patients.

Coagulin, a poly-protein complex consisting of coagulin I and coagulin II 12, plays a crucial role in chromosome assembly and separation during mitosis and meiosis 13. The non-structural maintenance of chromosomes (SMC) coagulin I complex H subunit (NCAPH) is encoded by a gene located on chromosome 2q11.2 14. Research indicates that NCAPH is implicated in the pathogenesis of various malignant tumors, impacting patient survival and prognosis. In hepatocellular carcinoma, elevated NCAPH expression levels have been observed compared to normal tissue, promoting tumor cell proliferation, migration and invasion 15. In serous ovarian cancer patients, the expression level of NCAPH is increased, and its high expression can cause chemotherapy resistance in patients 16. However, the function and pathogenesis of NCAPH in low-grade gliomas (LGG) have been poorly studied, so this study will systematically investigate the effect of NCAPH on LGG and its potential as a target for advancing the diagnosis and treatment of LGG.

**Figure 1:**
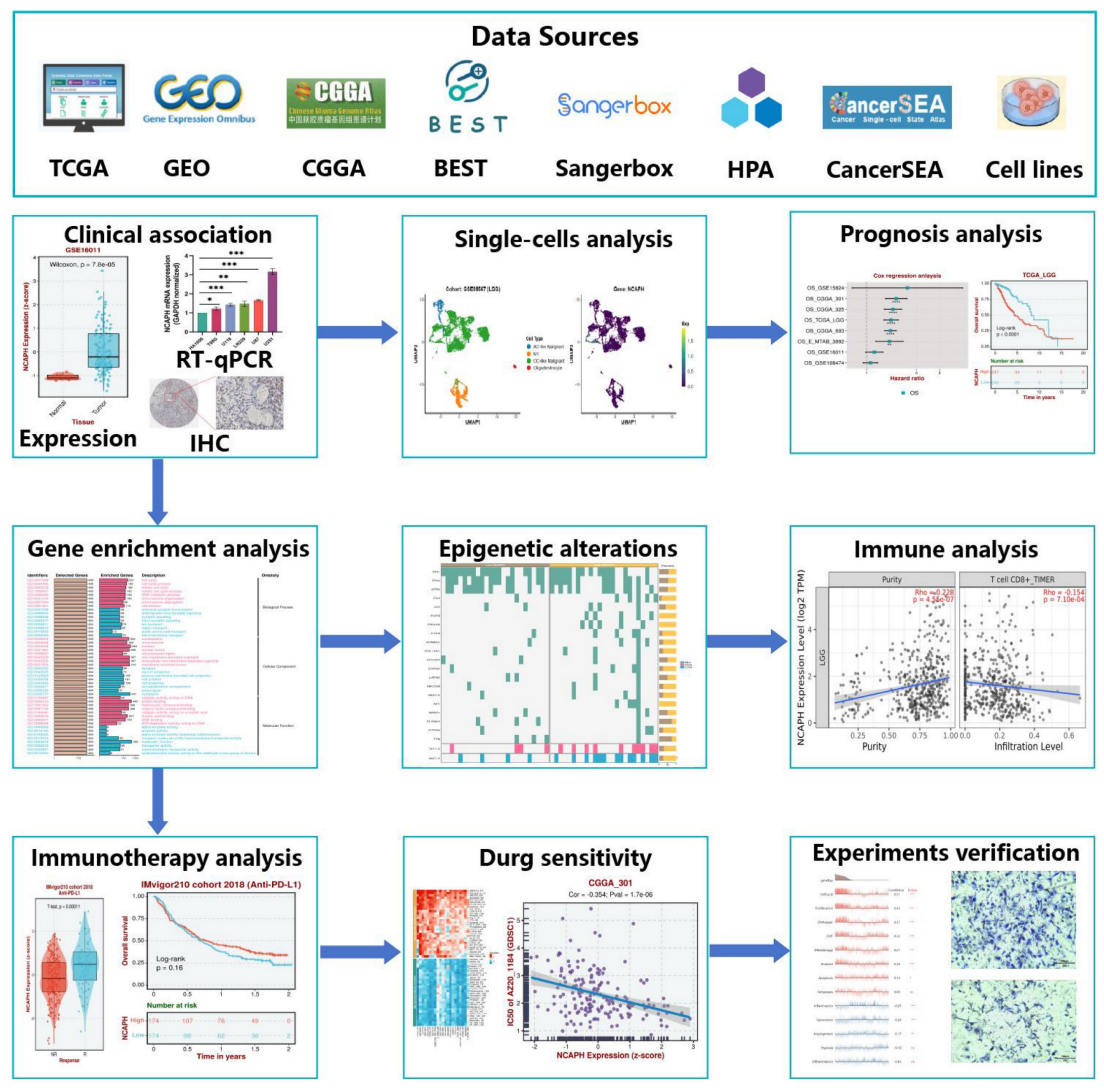
Workflow of the present study.

Here, this study attempts to reveal the expression level and single-cell subtype abundance of NCAPH in normal human tissues, pan-cancer, brain tumor cells, brain tumor tissues, and LGG, thereby suggesting the potential effect of NCAPH on LGG. This study will examine the effects of NCAPH on clinicopathological parameters, patient prognosis, biological function, epigenetic alterations, immune cell infiltration, immunotherapy, potential therapeutic drugs, and identify NCAPH as a potential immune-related therapeutic target for LGG patients (Figure 1).

## 2.Materials and Methods

### 2.1. TCGA, GTEx, CGGA and GEO datasets

Transcriptional expression data and related clinical information of NCAPH were gained from TCGA 17, GTEx 18, CGGA 19 and GEO 20 databases. After obtaining RNA-seq data from these databases, we converted it into TPM format and performed log2 conversion. The above data is from a public database and therefore does not require Ethics Committee approval.

### 2.2. RNA-sequencing analysis of NCAPH in pan-cancer

Firstly, NCAPH expression RNA-seq data of healthy human was obtained from GTEx database, and then NCAPH mRNA expression of different tumor cells and NCAPH expression of brain tumor cells were analyzed via HPA database 21. Finally, RNA-seq data based on TCGA database was applied to study the mRNA expression of NCAPH in pan-cancer.

### 2.3. Clinicopathological analysis of NCAPH in low-grade glioma

Based on CGGA database (CGGA_301, CGGA_325 and CGGA_693), we used BEST website (https://rookieutopia.com/app_direct/BEST/) 22 to analyze the association between NCAPH expression and tissue, type, grade and treatment in LGG. Once on the site, click on Step I: Select your Cancer Type - LGG, including 8 datasets and 1846 samples; Step II: Determine your Analysis Module - Single Gene - Go explore BEST, and then input NCAPH. We analysis different tissue groups, different type groups, different grades, different treatment groups in “Clinical association” section 23. Moreover, we analysis the NCAPH protein expression in normal and LGG patients based on HPA dataset.

### 2.4. Single‑cell analysis of NCAPH in low-grade glioma

The HPA database provides scRNAseq data that encompasses samples from 31 major health-related tissues and organs, as well as 557 individual cell type clusters. To avoid technical deviations and ensure that the single-cell datasets can best represent the corresponding tissues, we applied the following criteria for data selection: (1) Single-cell RNA sequencing was performed on the single-cell suspensions from tissues without pre-enrichment of cell types; (2) Datasets included >3,000 cells and 20 million read counts; (3) Pseudo-bulk gene expression profiles were highly correlated with bulk RNA-seq profiles. “Single cell” section of HPA database was used to explore the abundance and clustering of NCAPH in different brain cell subpopulations, and the GSE89567 dataset was used to further validate the abundance of NCAPH in each cell subpopulation in the LGG single-cell cohort 22, 24.

### 2.5. Quantitative real-time PCR (RT‒qPCR)

Five human glioma cells (U87, T98G, U118, LN229 and U251) and one normal human glioma cell HA1800 were selected to extract total RNA from the cells. The RNA was reverse-transcribed into cDNA using Takla's PrimeScript™ RT reagent Kit with gDNA Eraser, followed by amplification of the cDNA with TB Green® Premix Ex Taq™ II (Tli RNaseHPlus). The primer sequences utilized for NCAPH and GAPDH were as follows: NCAPH, forward primer, 5′-AAACACGCAGATTACGGAACA-3′, reverse primer, 5′-GTTGGTTGGTTCGGTGTCTTT-3′; GAPDH, forward primer, 5'-CAGGAG GCATTGCTGATGAT-3', reverse primer, 5'-GAAGGCTGGGGCTCATTT-3'.

### 2.6. The prognostic value of NCAPH in low-grade glioma

Survival data for LGG were collected from multiple datasets including TCGA, CGGA, and GEO. Kaplan-Meier analysis was utilized to examine patients' overall survival (OS), disease-specific survival (DSS), disease-free interval survival (DFI), and progression-free interval (PFI). Nomogram plots were employed to predict the impact of NCAPH and other clinical parameters on survival outcomes in LGG patients, while calibration plots were utilized to evaluate 1-year, 3-year, and 5-year overall survival rates in this patient population.

### 2.7. PPI networks and functional enrichment analysis

The protein-protein interaction (PPI) analysis database STRING 25 helps us to obtain gene networks that are positively and negatively correlated with NCAPH. GO and KEGG enrichment analyses specifically analyzed the biological function of genes co-expressed with NCAPH, the "ClusterProfiler" package was used for these analyses, and the consequences were visualized via the "ggplot2" package. For gene enrichment analysis (GSEA), pearson correlation coefficient was used to calculate the genes associated with NCAPH (P<0.05). All analyses were performed using DAVID Bioinformatics Resource 6.8 (https://davidbioinformatics.nih.gov/) 26.

### 2.8. Correlation analysis of NCAPH expression with immune infiltration

The research investigated the relationship between NCAPH expression and immune cell infiltration in lower grade gliomas (LGG) by analyzing data from the "Cell infiltration" section of the BEST database. The study utilized the R package "limma" to determine the correlation between NCAPH expression and various immune-related genes, including immunostimulatory genes, immunosuppressive genes, chemokine receptors, chemokine genes, and antigen-presenting related genes. The results were visualized as heat maps using the R-package "heatmap." Furthermore, the study delved into the association between NCAPH expression and tumor infiltrating immune cells (TIICs) in LGG. using the TIMER, EPIC, MCPCOUNTER, CIBERSORT, CIBERSORT-ABS, QUANTISEQ, XCELL and ESTIMATE algorithms.

### 2.9. Immunotherapy response assessment and drug sensitivity analysis

In order to explore the immunotherapy response of LGG patients with different subtypes, we collected three immunotherapy cohorts, including IMvigor210 cohort 2018, Hugo cohort 2016 and Cho cohort 2020, based on the BEST database. Anti-PD-L1, Anti-PD-1 and Anti-PD-L1 /PD-1 immunotherapy approaches were applied, respectively, to identify subtypes with better immunotherapy responses. Sensitivity scores for each molecular compound were evaluated using the R-package "pRRophetic" to obtain the eight drugs that were most sensitive and tolerated to LGG patients with high NCAPH expression.

### 2.10. Candidate agents of NCAPH in LGG and molecular docking

Drug response data was obtained from BEST website based on the following databases: GDSC (https://www.cancerrxgene.org/) 27, CTRP (https://portals.broadinstitute.org/ctrp/) 28, and PRISM (https://depmap.org/portal/prism/) 29. In order to explore the correlation between NCAPH expression and the IC50 of candidate agents, we selected 8 datasets. The top ten candidate agents that positive and negative correlation with NCAPH were chosen. We selected 4 drugs that are sensitive to LGG patients with high expression of NCAPH, downloaded their molecular formulas from PubChem website (https://pubchem.ncbi.nlm.nih.gov/) 30, and analyzed their conformation and affinity in proteins using Protein data bank website (https://www.rcsb.org/) 31.

### 2.11. Cell counting Kit‑8 assay (CCK8)

CCK8 assays were performed using U251 and LGG cell line SW1088 to assess the influence of NCAPH expression on cell viability. CCK8 reagents were purchased from AbMole BioScience, USA (Cat: M4839-500Tests). We transfected cells with siNCAPH, digested the cells with trypsin 24 hours later, and then spread them into 96-well plates with 1000 cells per well. CCK8 reagent was added at 0h, 24h, 48h, 72h and 96h, respectively, with 10 microliters per well. Our final step was to analyze the results using statistical methods after measuring the absorbance of each well for two hours using a spectrophotometer. (**** p<0.0001).

### 2.12. Invasion assay (Transwell)

For the invasion assay, 20,000 cells were placed in the upper layer of Matrigel coating, the lower layer was added with DMEM including 10% serum, and cultured in an incubator for 24 hours. Fixing the cells with methanol, staining them with 0.1% crystal violet for 30 minutes, gentle washing with PBS, and examining them under a microscope were the next steps. No less than three areas were randomly selected for each chamber to image the cells.

### 2.13. Statistical analysis

Data in this study were analyzed using GraphPad Prism 10 and R software. Differential expression was assessed with T test, Wilcoxon test, or Two-way ANOVA, while prognostic analysis used the log-rank test. Spearman correlation was used to determine correlation between groups. The difference in NCAPH mRNA expression was evaluated with Student's t test on PR-qPCR data. Significance levels were denoted as *: p < 0.05, **: p < 0.01, ***: p < 0.001.

## 3. Results

### 3.1. NCAPH is significantly highly expressed in several cancer types

**Figure 2:**
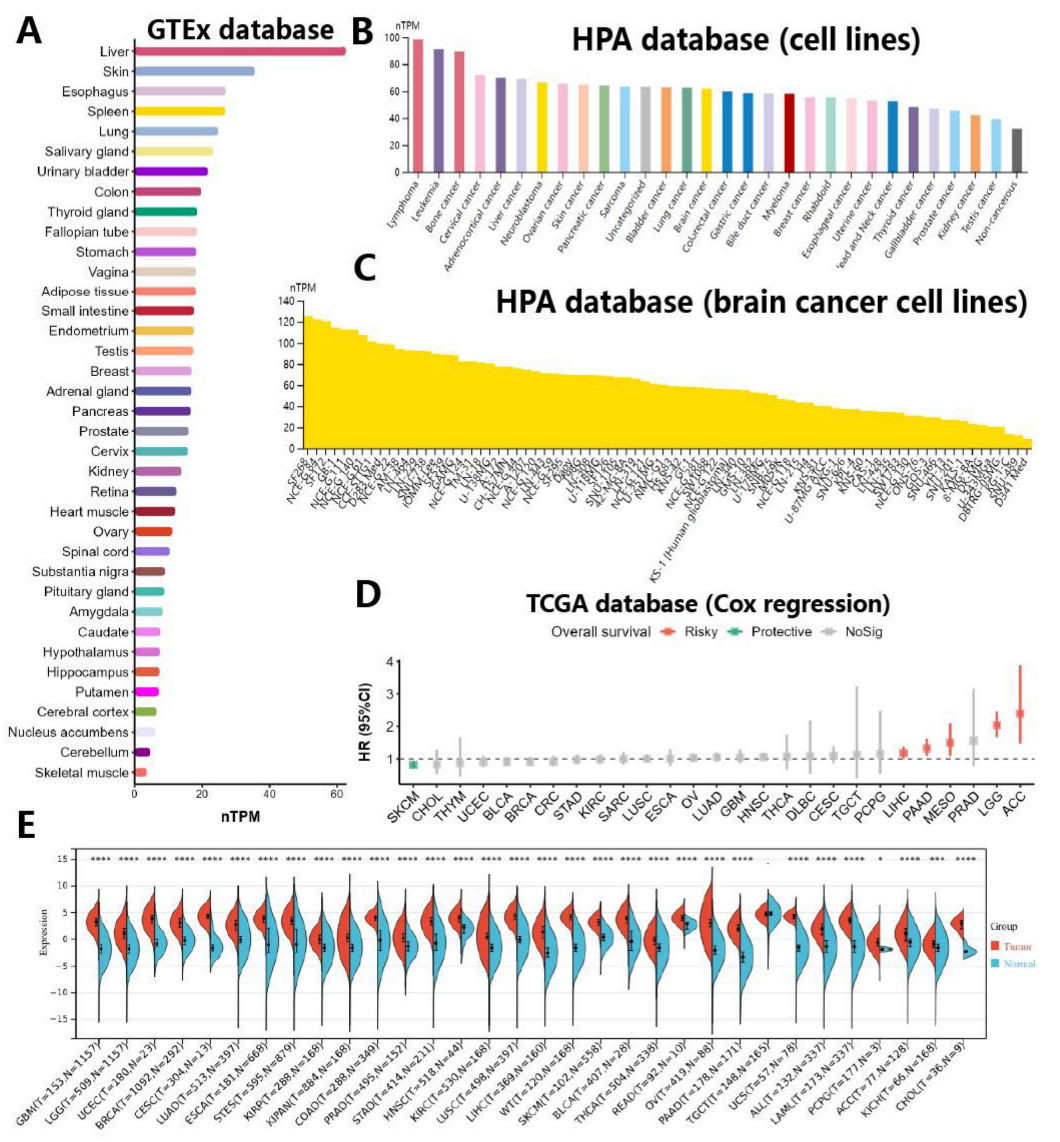
NCAPH expression in different types of cell lines, tissues and cancers. **A** Differences in NCAPH expression in normal tissues in GTEx database. **B** The overall differences of NCAPH expression in cancer cell lines in HPA database. **C** Differences in NCAPH expression in brain cancer cell lines in HPA dataset. **D** Prognosis and risk assessment of NCAPH in cancer patients in TCGA database. Red indicates risky, and green indicates protective. **E** Pan-cancer analysis of NCAPH in TCGA and GTEx databases. * P<0.05, ** P<0.01, *** P<0.001, **** P<0.0001.

In order to examine the variance in NCAPH expression across human normal tissues, cancer tissues, and cells, we conducted an analysis utilizing the TCGA, GTEx, and HPA databases to assess RNA sequencing data from a range of tissues and diseases. Our findings indicated that NCAPH exhibited elevated expression levels in normal liver, skin, and esophagus, with notably low expression levels observed in normal brain tissue (Figure 2A). Moreover, analysis of the HPA database revealed that NCAPH was predominantly expressed at high levels in lymphoma, leukemia, and bone cancer, while its expression in brain tumors was moderate (Figure 2B). In brain tumor cells, SF268, CNE-G84 and SF172 were highly expressed, while D341 Med, SNU-489 and KG-1-C were less expressed (Figure 2C). The examination of overall survival rates (OS) across different types of cancers indicated that elevated NCAPH expression posed a risk for adrenal cortical carcinoma (ACC), low-grade glioma (LGG), mesothelioma (MESO), pancreatic cancer (PAAD), and hepatocellular carcinoma (LIHC), while serving as a protective factor for cutaneous melanoma (SKCM) (Figure 2D). Additionally, a comprehensive analysis of 32 cancers and their corresponding adjacent tissues demonstrated that NCAPH exhibited high expression levels in the majority of cancers, notably in LGG (Figure 2E).

Our study shows that NCAPH is low expressed in normal brain tissue and high expressed in brain tumors, especially LGG, and that NCAPH may be a potential risk factor in LGG patients.

### 3.2. Relationship between NCAPH expression and clinicopathologic feature in LGG

**Figure 3:**
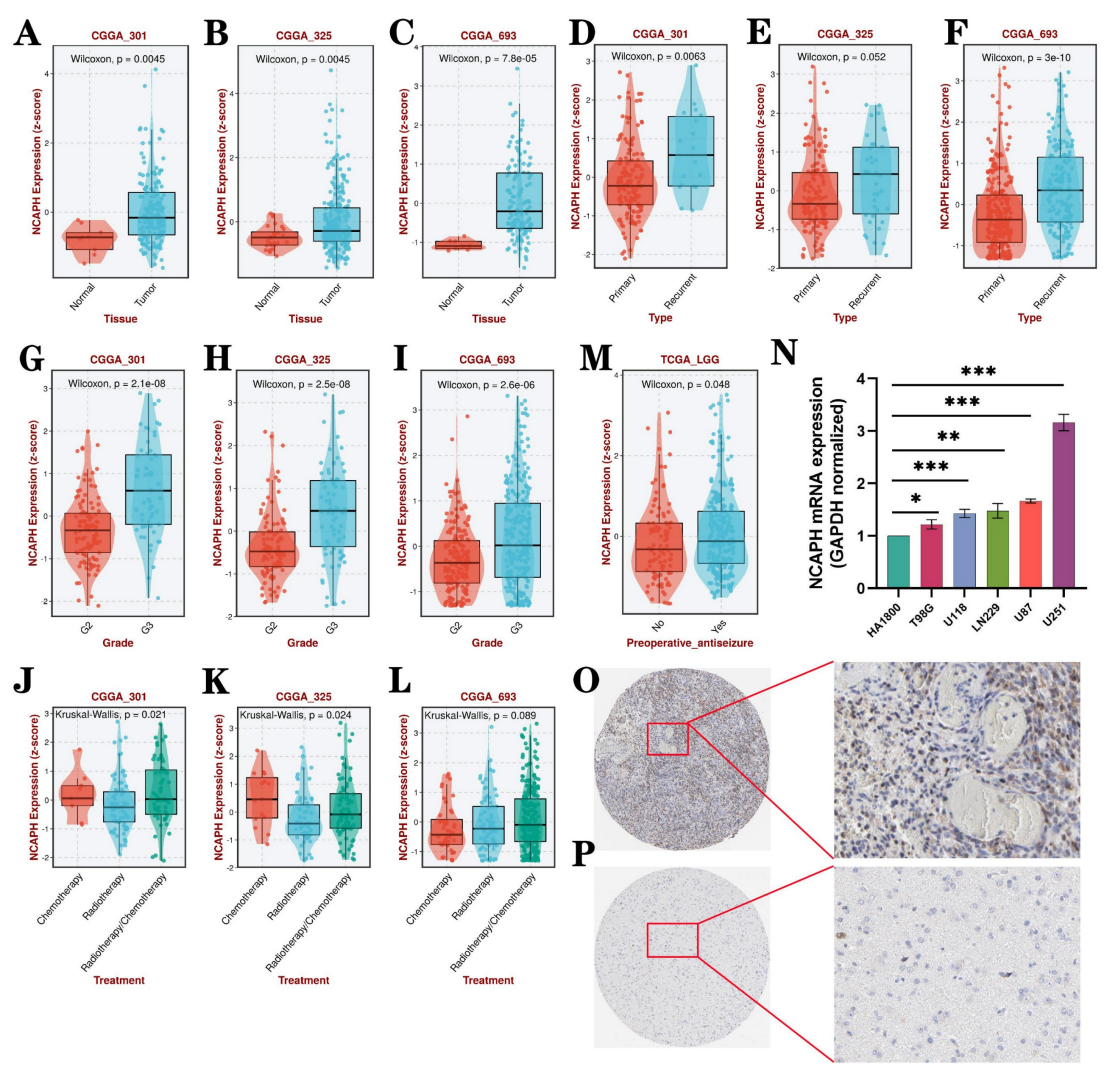
Relationship between NCAPH expression and clinicopathological characteristics. NCAPH expression was significantly relevant to the following factors: **A-C** tissue, **D-F** type, **G-I** grade, **J-L** treatment, **M** preoperative antiseizure. **N** NCAPH mRNA expression in 5 glioma cell lines and 1 glial cell line using RT-qPCR.** O-P** Based on HPA dateset, the expression of NCAPH protein in glioma and normal tissues using IHC assay (*P < 0.05, **P < 0.01, ***P < 0.001).

The above study results preliminatively showed NCAPH expression through TCGA, GTEx and various cell lines. And then we analyzed the clinicopathological features of NCAPH in LGG by using the three CGGA datasets (CGGA_301, CGGA_325 and CGGA_693). The study indicates a significant upregulation of NCAPH mRNA expression in LGG tissues compared to normal controls (Figure 3A-C). Moreover, NCAPH expression was found to be elevated in relapsed patients compared to those newly diagnosed (Figure 3D-F), and showed a positive correlation with LGG grade (Figure 3G-I). Additionally, our analysis revealed lower NCAPH expression in the radiotherapy group compared to the chemotherapy and chemoradiotherapy groups, particularly in the CGGA_301 and CGGA_325 datasets (Figure 3J-L). These results suggest that LGG patients with elevated NCAPH levels may exhibit heightened sensitivity to radiotherapy. Interestingly, it was observed that preoperative antiseizure patients with low-grade gliomas exhibited elevated levels of NCAPH expression compared to the untreated cohort, as depicted in Figure 3M. Subsequently, RT-qPCR assays were conducted on five glioma cell lines and one glial cell line, as shown in Figure 3N, while immunohistochemical (IHC) experiments were carried out on glioma tissues and normal tissues, as illustrated in Figure 3O-P. These findings collectively support the notion that both mRNA and protein expression levels of NCAPH in gliomas surpass those in the normal control group.

Following this, data was gathered from 532 LGG patients in the TCGA database to evaluate potential risk factors for this patient population. Univariate and multivariate Cox regression analyses demonstrated that several factors, including WHO grade, IDH status, primary therapy outcome, age, and NCAPH expression level, significantly influenced the survival and prognosis of patients with LGG. In contrast, variables such as 1p/19q codeletion, gender, and histological type did not exhibit statistical significance in relation to the risk of LGG patients. Of particular note, elevated levels of NCAPH expression were found to be associated with a heightened risk for LGG patients, as indicated in Table 1.

### 3.3. Expression abundance of NCAPH in each cell subpopulation of the LGG single-cell cohort

**Figure 4:**
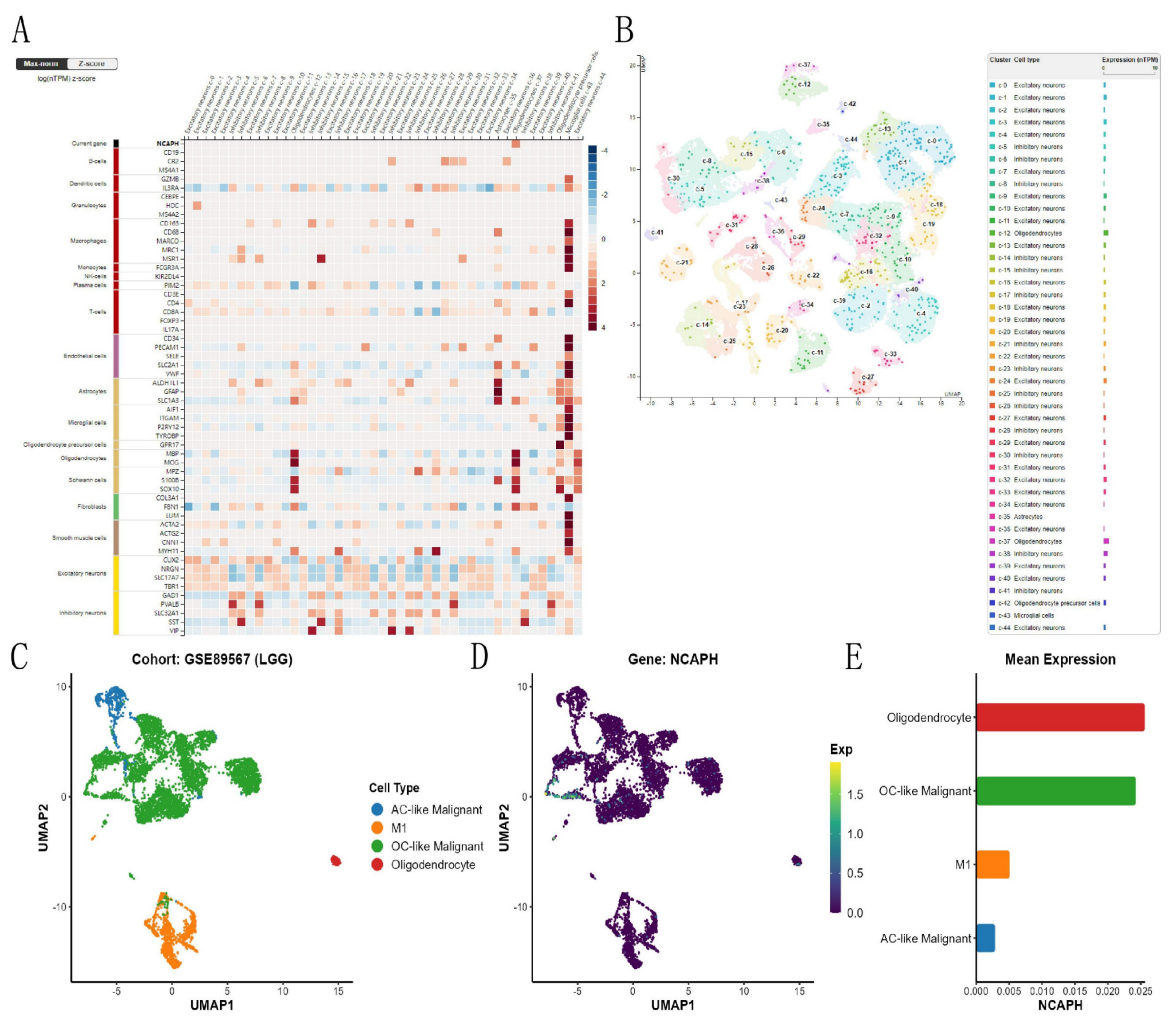
Single-cell analysis of NCAPH in normal brain tissues and LGG patient tissues. **A-B** Expression and clustering of NCAPH in normal brain tissue; **C-E** Expression and clustering of NCAPH in LGG patients' tissue.

Based on the HPA database, we studied the expression and distribution of NCAPH in different neuronal cells and glial cells of the brain, and the consequences revealed that NCAPH was mainly expressed in the oligodendrocytes of the brain (Figure 4A-B). Then, we conducted UMAP analysis of NCAPH expression in LGG based on the GSE89567, and the consequences revealed that the main NCAPH expression cells in LGG were AC-like malignant cells, M1, and OC-like malignant cells and oligodendrocytes, and only oligodendrocytes and OC-like malignant cells had high expression levels (Figure 4C-E).

### 3.4. Co-localization analysis and prognosis analysis of NCAPH in glioma

**Figure 5:**
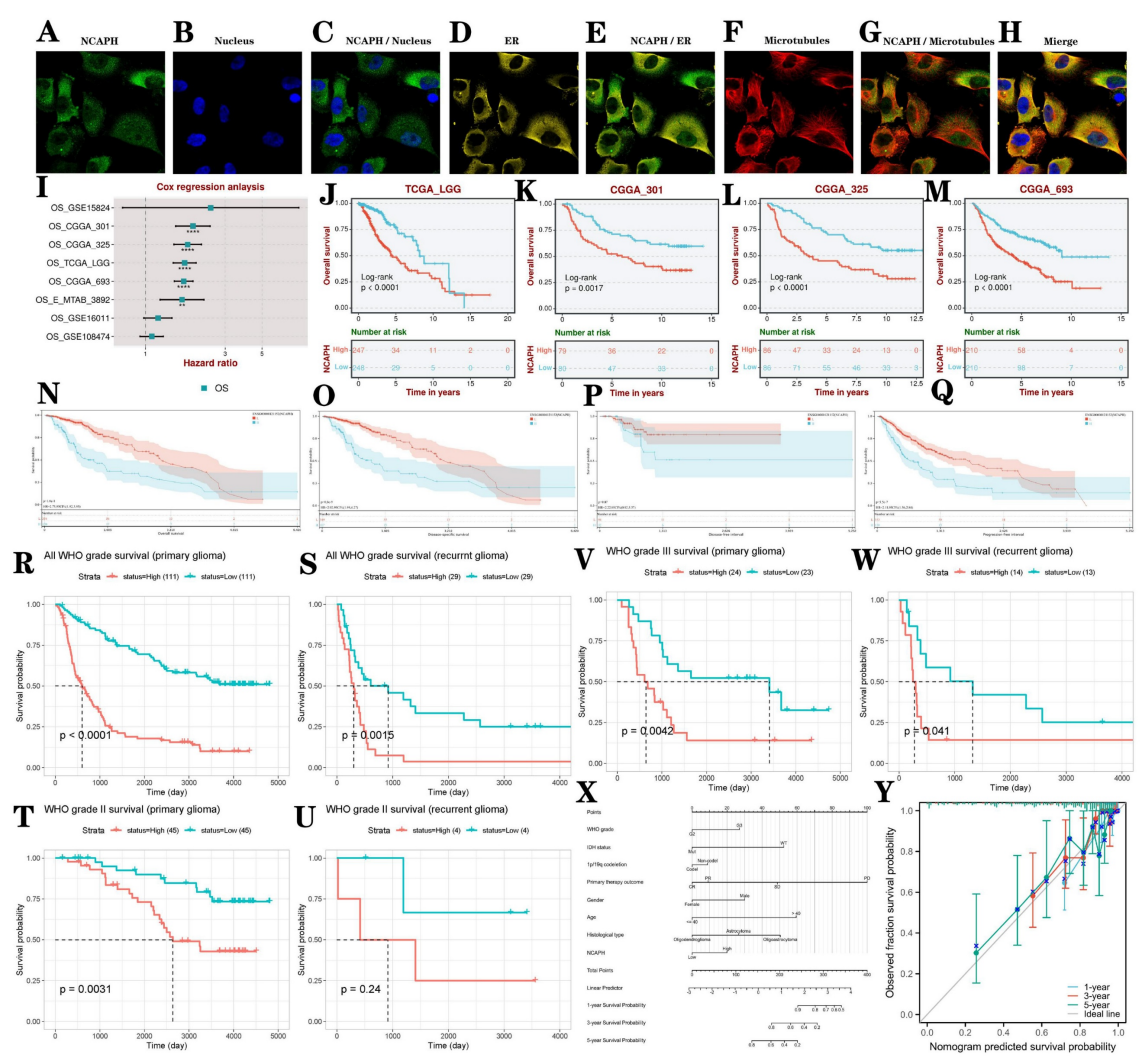
The study examines the diagnostic and prognostic significance of NCAPH in LGG. **A-H** Colocalization analysis of NCAPH in U251 cells is conducted. **I-M** The impact of NCAPH expression on the prognosis of LGG patients is evaluated using data from TCGA, CGGA, and GEO databases. **N-Q** The effects of NCAPH expression on OS, DSS, DFI, and PFI in LGG patients are analyzed based on data from TCGA and GTEx databases. **R-W** the influence of NCAPH expression on the prognosis of primary and recurrent LGG patients is investigated using data from the CGGA database. **X** a nomogram is developed that integrates risk score with NCAPH expression, WHO grade, IDH status, primary therapy outcome, age, 1p/19q codeletion status, gender, and histological type. **Y** Calibration plots for predicting 1-, 3-, and 5-year OS of LGG patients.

Co-localization of proteins or molecules within cells holds significant biological importance. It can reveal the functions of proteins/molecules, help understand the dynamics and interactions of organelles, assist in studying the mechanisms of disease occurrence, guide experimental design and drug development 32. We evaluated the distribution of NCAPH in the nucleus, microtubules, and endoplasmic reticulum (ER) of the U251 cell line, showing that NCAPH is primarily colocalized with microtubules and ER (Figure 5A-H). We conducted a systematic analysis to investigate the influence of elevated NCAPH expression on the survival and prognosis of LGG patients using data from TCGA, CGGA, and GEO datasets. Our findings revealed that increased NCAPH expression was relevant to a significant decrease in overall survival among patients (Figure 5I-M). Furthermore, we examined the influence of NCAPH expression levels on various survival parameters, including overall survival (OS), disease-specific survival (DSS), disease-free interval (DFI), and progression-free interval (PFI) in LGG patients using data from TCGA and GTEx datasets. The consequences demonstrated that high NCAPH expression was correlated with a significant reduction in OS, DSS, and PFI in LGG patients (Figure 5N-Q). Subsequently, an examination was conducted to evaluate the influence of NCAPH expression levels on the prognostic outcomes of primary and recurrent glioma patients using data from CGGA. The findings indicated that individuals with elevated NCAPH expression levels, particularly those diagnosed with WHO grade III glioma, exhibited unfavorable prognoses in both primary and recurrent cases (Figure 5R-W). Therefore, it can be concluded that patients with LGG and heightened NCAPH expression levels are associated with poorer prognostic outcomes.

In order to conduct a comprehensive evaluation of the clinicopathological characteristics and prognostic impact of NCAPH in patients with LGG, a column graph was created to incorporate variables such as NCAPH expression level, WHO grade, IDH status, primary therapy outcome, age, 1p/19q codeletion, gender, and histological type. These factors were utilized to analyze the survival rates at 1, 3, and 5 years in patients with LGG (Figure 5X). Additionally, a calibration curve was developed to assess the accuracy of the nomogram in predicting survival outcomes in patients with LGG (Figure 5Y).

### 3.5. PPI networks and gene enrichment analysis

**Figure 6:**
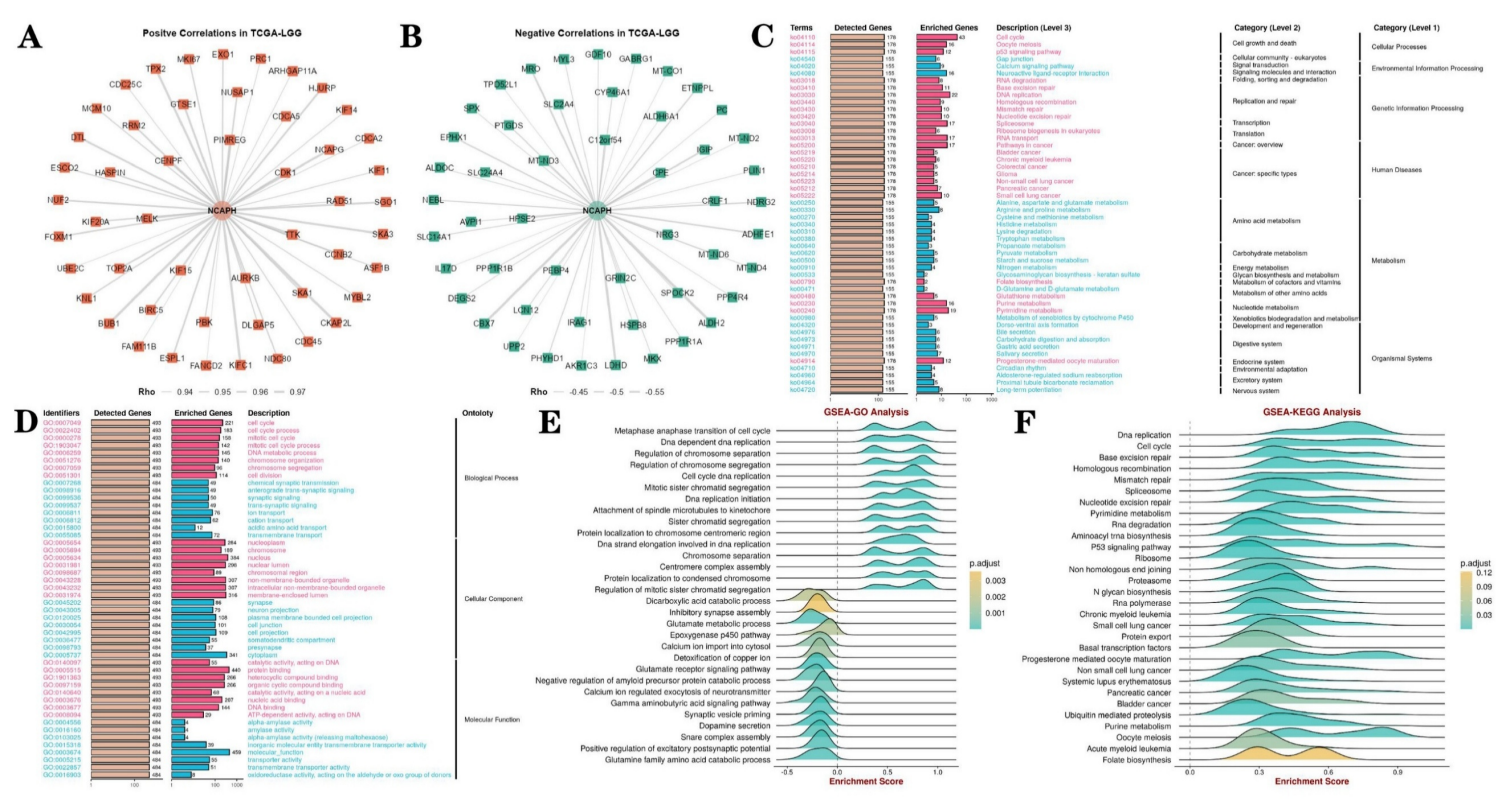
PPI networks and GO, KEGG and GSEA analysis. **A-B** The PPI network of NCAPH and the top 50 positive co-expressed genes and the top 50 negative co-expressed genes. **C-D** GO and KEGG pathway enrichment analyses, including biological process, cellular component, molecular function, cellular processes, environmental information processing, genetic information processing, human disease, metabolism, organismal systems.** E-F** GSEA is further used to analyze the potential functions of NCAPH.

Protein interaction network analysis revealed 100 kinds of proteins positively and negatively correlated with NCAPH, among which the positive correlated proteins mainly included CENPF, PIMREG, CDK1, AURKB and TTK, while the negative correlated proteins mainly included PEBP4, GRIN2C, NRG3, CPE and HPSE2 (Figure 6A-B). Then, we conducted GO and KEGG enrichment analysis of NCAPH related genes, and the results showed that NCAPH may be closely related to cell cycle, DNA replication, membrane-enclosed lumen, protein binding and molecular function. GSEA further found that NCAPH is associated with metaphase anaphase transition of cell cycle, DNA strand elongation involved in DNA replication, glutamate receptor signaling pathway, cell cycle, DNA replication and base excision repair.

### 3.6. Epigenetic alterations of NCAPH in different tumors, especially LGG

**Figure 7:**
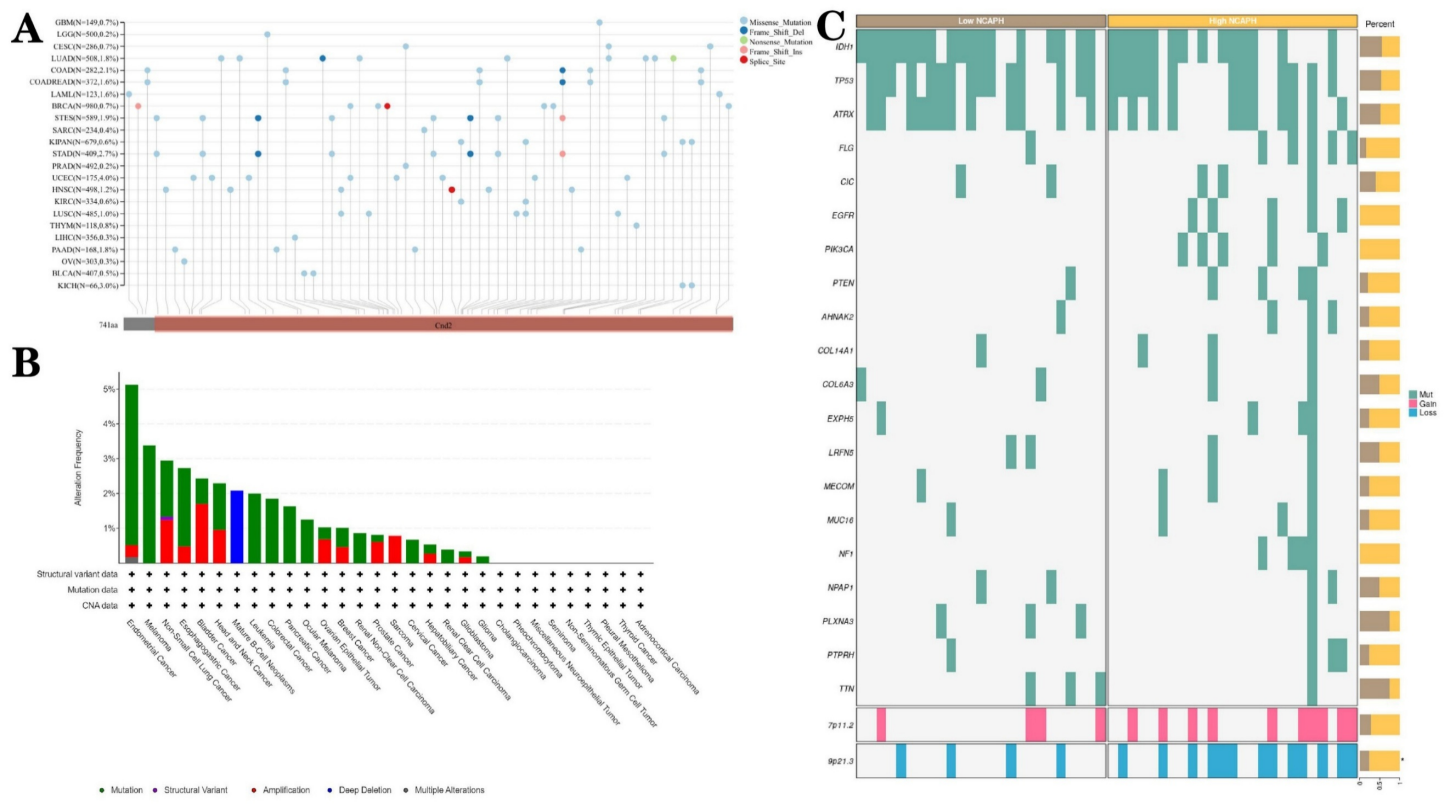
Genetic alterations of NCAPH in pan-cancer and LGG. **A-B** Mutant landscape of NCAPH in pan-cancer;** (C)** Mutant landscape of NCAPH in LGG.

The nucleotide variation dataset of TCGA samples was obtained from the GDC database after processing by the MuTect2 software 33. Integration of mutation data and retrieval of protein domain information were performed using the R software package maftools. Analysis indicated that missense mutations were the predominant epigenetic alterations in LGG (Figure 7A). Given the importance and frequency of genetic mutations in tumorigenesis, a comprehensive analysis of the mutation landscape of NCAPH across various cancer types was conducted using the cBioPortal database, encompassing data from 10,953 patients. The results revealed that missense mutations, amplification, and deep deletion were the predominant forms of NCAPH mutations, with endometrial cancer, melanoma, and non-small cell lung cancer being the most frequently mutated cancer types (Figure 7B). Furthermore, patients with LGG exhibiting high NCAPH expression were found to have a higher likelihood of 7p11.2 chromosome duplication and 9p21.3 chromosome deletion (Figure 7C).

### 3.7. NCAPH expression is markedly associated with tumor microenvironment, immune cell infiltration and immunoregulation‑related genes

**Figure 8:**
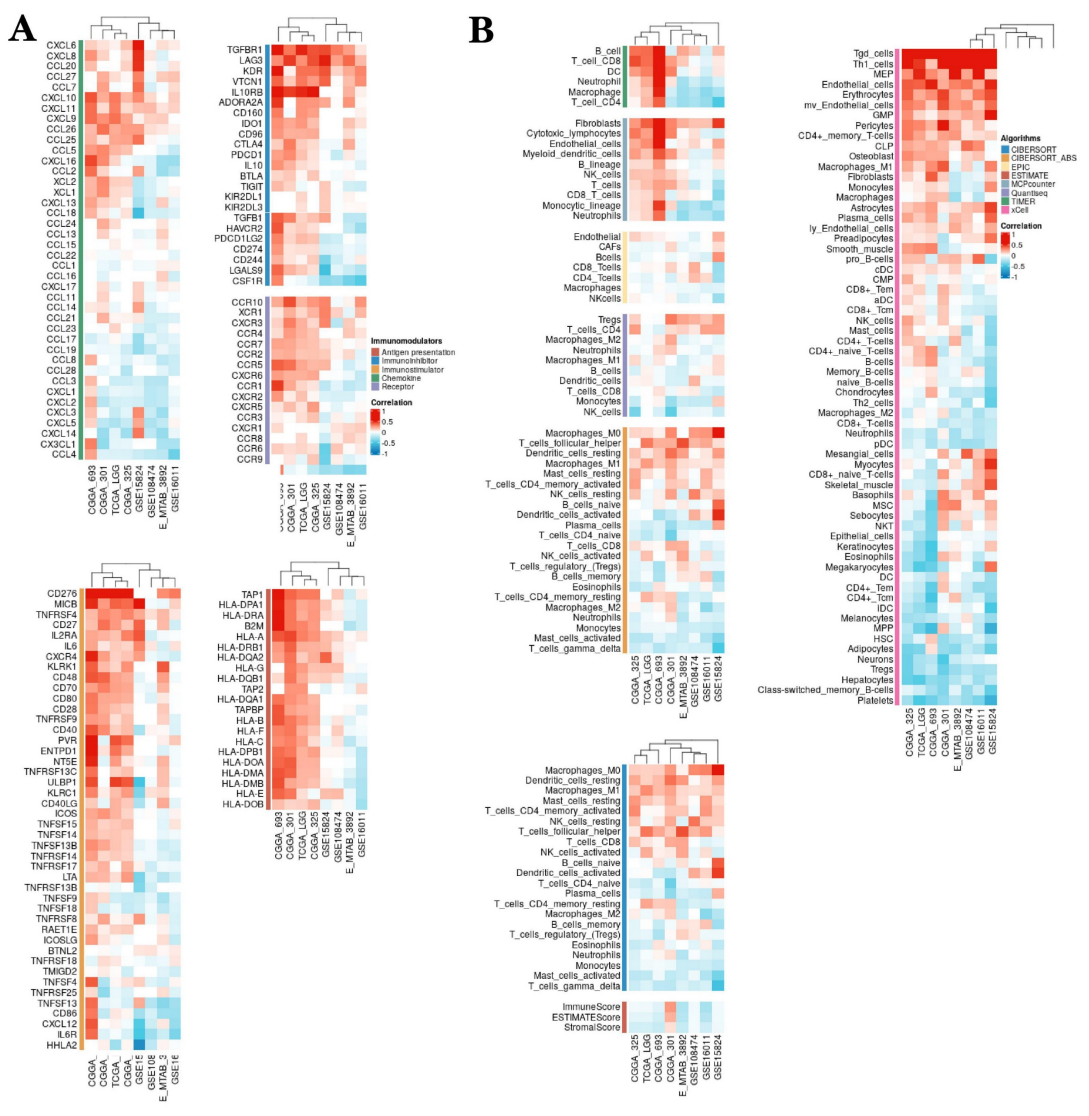
The relationship between NCAPH expression and immune cell infiltration and immunoregulation-related genes.** A** The relationship between NCAPH expression and immune-activating genes, immunosuppression-associated genes, chemokine receptors and chemokines based on multiple datasets.** B** The correlation of NCAPH and immune cell infiltration levels based on different algorithms.

The tumor microenvironment (TME) is a complex ecosystem that encompasses various components including tumor cells, tumor stem cells, stromal cells, microvessels, lymphatic vessels, and extracellular matrix. This environment plays a crucial role in promoting the proliferation, invasion, and metastasis of tumor cells. Within the TME, the tumor immune microenvironment (TIME) is a sophisticated framework characterized by the presence of diverse immune cells such as T cells, B cells, NK cells, DC cells, and Treg cells, as well as immune regulatory molecules like TGF-β 34. Research has indicated a strong correlation between the expression of NCAPH and TIME. To further explore this relationship, we studied the connection of NCAPH with immune checkpoint genes, immunostimulatory genes, immunosuppressive genes, chemokine receptors, and chemokines, as well as their implications for LGG immunotherapy using data from TCGA, CGGA, and multiple GEO datasets. Our findings revealed a positive association between the expression of NCAPH in LGG and immunosuppressive genes TGFBR1, LAG3, and KDR, a positive correlation with immunostimulatory genes CD276, TNFRSF4, and MICB, and a negative correlation with chemokines CCL4, CX3CL1, CXCL14 and CXCL5, and negatively correlated with immunosuppressive genes HHLA2, IL6R and CXCL12 (Figure 8A). Therefore, our study suggests that NCAPH may serve as an immune checkpoint for LGG, laying the foundation for further research on NCAPH immunotherapy in LGG.

We used a variety of algorithms, including CIBERSORT, EPIC, ESTIMATE, MCPcounter, TIMER, Quantlseq and xCELL, to assess the relevance between NCAPH expression and immune cell infiltration. The consequences showed that NCAPH in LGG was mainly positively correlated with Tgd cells, MEP, endothelial cells, erythrocytes, fibroblasts, Macrophages-M0 and Tregs cells, while negatively associated with NK cells, Gamma Delta T cells, Platelets, class-switched memory B cells, hepatocytes, neurons, monocytes and neutrophils (Figure 8B). These results further prove that the high NCAPH expression accelerates the formation of immunosuppressive TME.

### 3.8. Explore the relationship between NCAPH expression level and immunotherapy

**Figure 9:**
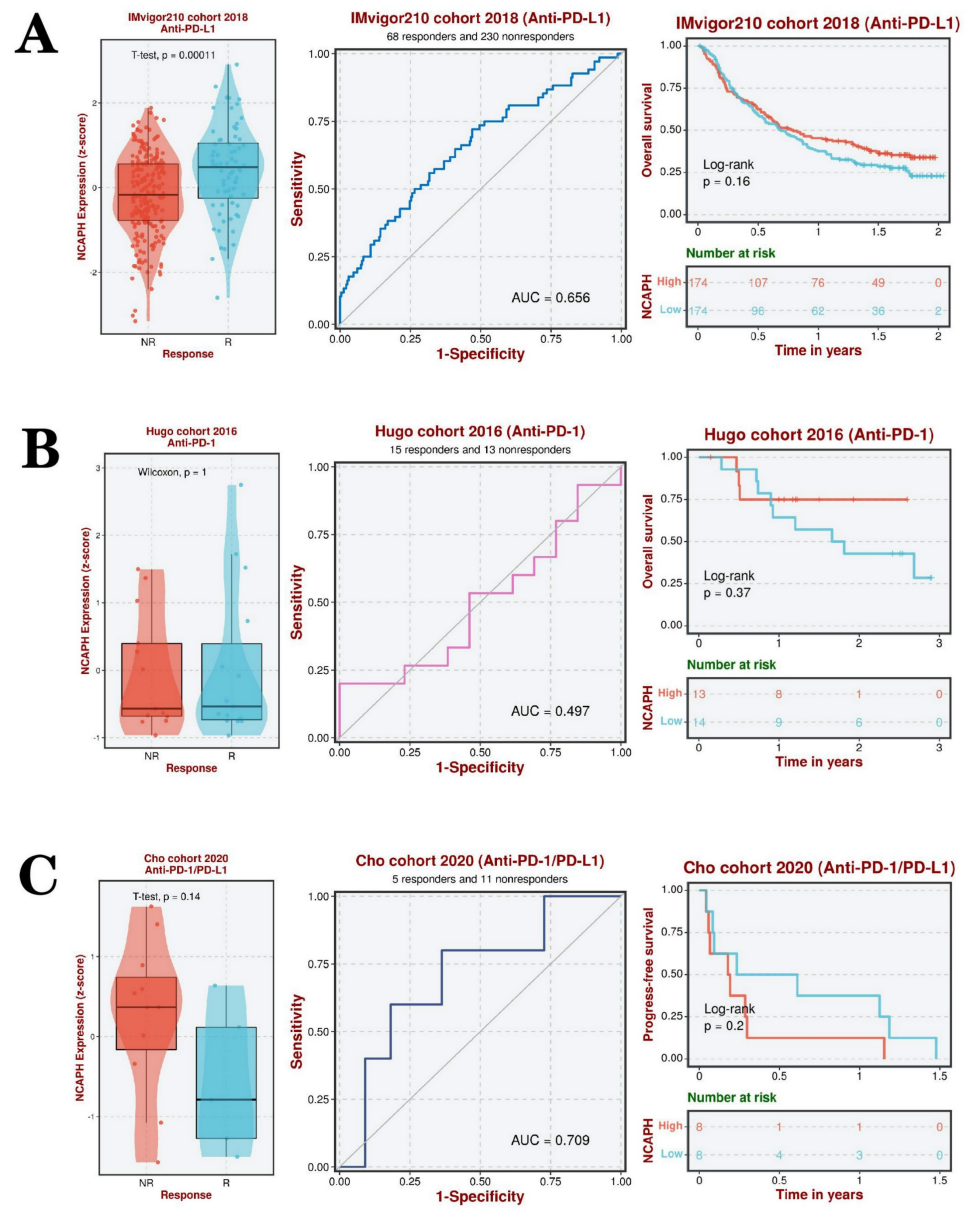
Analysis of association between NCAPH expression and immunotherapy and drug selection based on IMvigor210 cohort 2018, Hugo cohort 2016 and Cho cohort 2020 datasets. **A-C** Analysis of NCAPH expression levels and predictive value in LGG patients who respond and do not respond to anti-PD-L1, anti-PD-1, and anti-PD-L1 / PD-1 treatments, respectively; overall survival of LGG patients in the high and low expression NCAPH groups after anti-PD-L1, anti-PD-1, and anti-PD-L1 / PD-1 treatment, respectively (R, response; NR, non-response; AUC, area under curve).

The aforementioned study showed a significant positive correlation between the expression level of NCAPH and the immune checkpoint CD276. Utilizing three datasets (IMvigor210 cohort 2018, Hugo cohort 2016, and Cho cohort 2020), we conducted further investigations into the impact of anti-PD-L1, anti-PD-1, and anti-PD-L1/PD-1 therapies on the survival and prognosis of LGG patients, as well as the involvement of NCAPH in this process. The findings indicated that LGG patients who exhibited a favorable response to anti-PD-L1 treatment displayed elevated levels of NCAPH expression, with an area under the ROC curve of 0.656, suggesting a certain degree of predictive value. The research revealed that individuals exhibiting elevated levels of NCAPH demonstrated a marginally increased rate of survival in comparison to those with lower expression levels, albeit the disparity did not reach statistical significance (Figure 9A). Furthermore, there was no substantial difference in the levels of NCAPH expression between the anti-PD-1 group and the anti-PD-L1 /PD-1 group, and no notable variance in therapeutic effectiveness was observed (Figure 9B-C). These findings suggest that anti-PD-L1 therapy may be beneficial for patients with high NCAPH expression, while there is no discernible distinction in therapeutic outcomes between anti-PD-1 and anti-PD-L1 /PD-1 treatments.

### 3.9. Drug sensitivity analysis and molecular docking analysis

**Figure 10:**
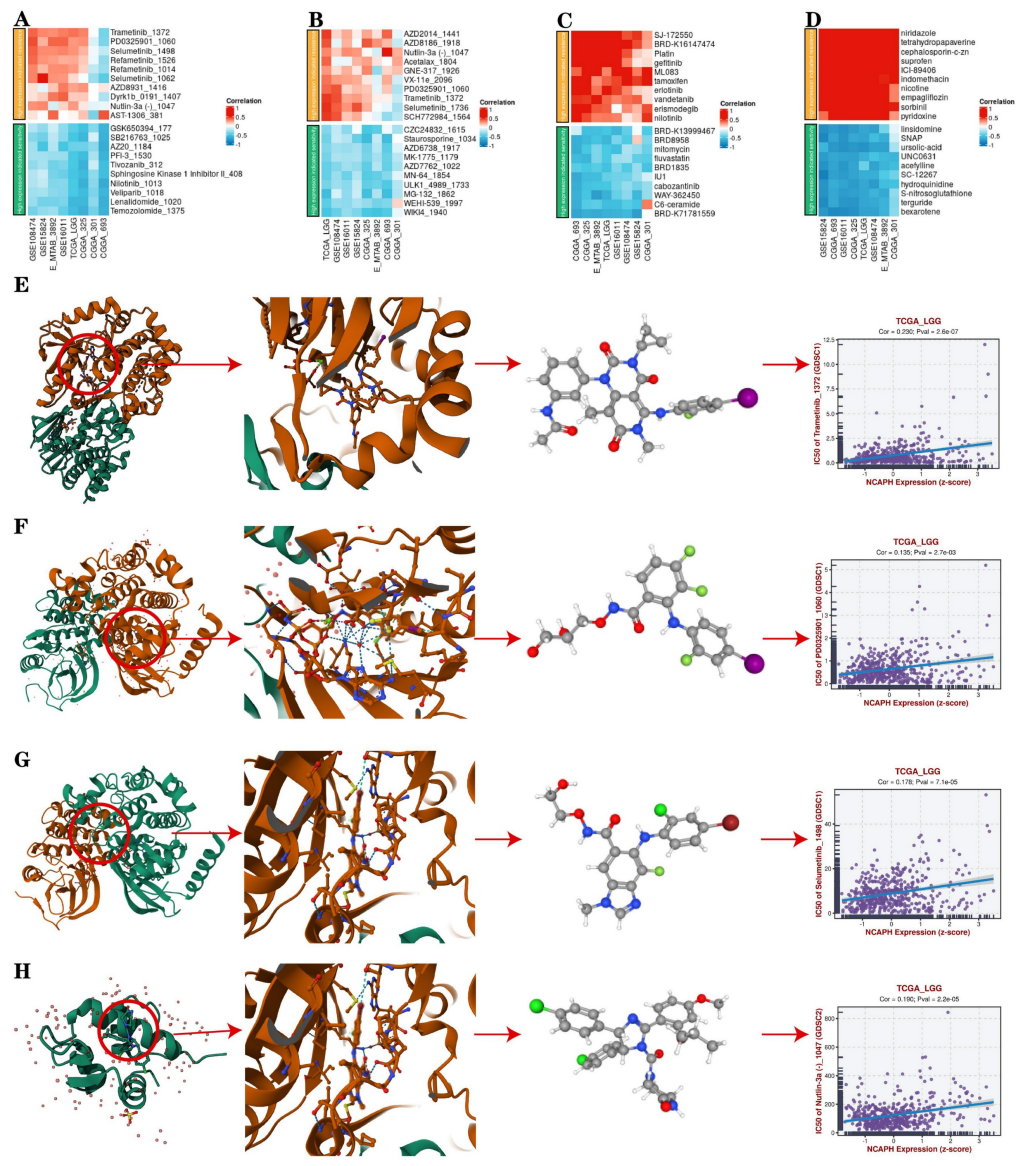
Drug sensitivity analysis and molecular docking analysis.** A-D** IC 50 value of 10 representative drugs that indicate resistance and sensitivity, respectively.** E-H** Drug sensitivity analysis and molecular docking analysis of Trametinib, Selumetinib, Nutlin-3a and PD0325901.

Furthermore, we conducted a drug sensitivity analysis of LGG patients with high expression of NCAPH based on the TCGA database, aiming to provide potential treatment options for such individuals, and found that LGG patients with high NCAPH expression were most sensitive to temozolomide, lenalidomide, SB216763 and AZ20, while most resistant to Trametinib, Selumetinib, Nutlin-3a and PD0325901 (Figure 10A-D). It is mentioned that temozolomide is currently the first-line treatment for LGG patients 35, the above consequences suggest that LGG patients with high NCAPH expression are sensitive to the treatment of temozolomide. And our analysis also provides researchers with ideas for other treatment sensitive drugs for LGG in the future, such as GSK-3 inhibitor SB216763 and ATR inhibitor AZ20.

In order to evaluate whether the above drugs can stably bind target proteins under physiological conditions, we selected Trametinib, Selumetinib, Nutlin-3a and PD0325901 for molecular docking analysis and visualization with corresponding target proteins. We found that these drugs can easily bind to these targets and have a relatively stable conformation, and the core target is closely related to tumor development and may be closely associated with the cancer-promoting effect of high expression of NCAPH (Figure 10E-H).

### 3.10. Knockdown NCAPH attenuates the proliferation and invasion of glioma cells and promotes apoptosis

**Figure 11:**
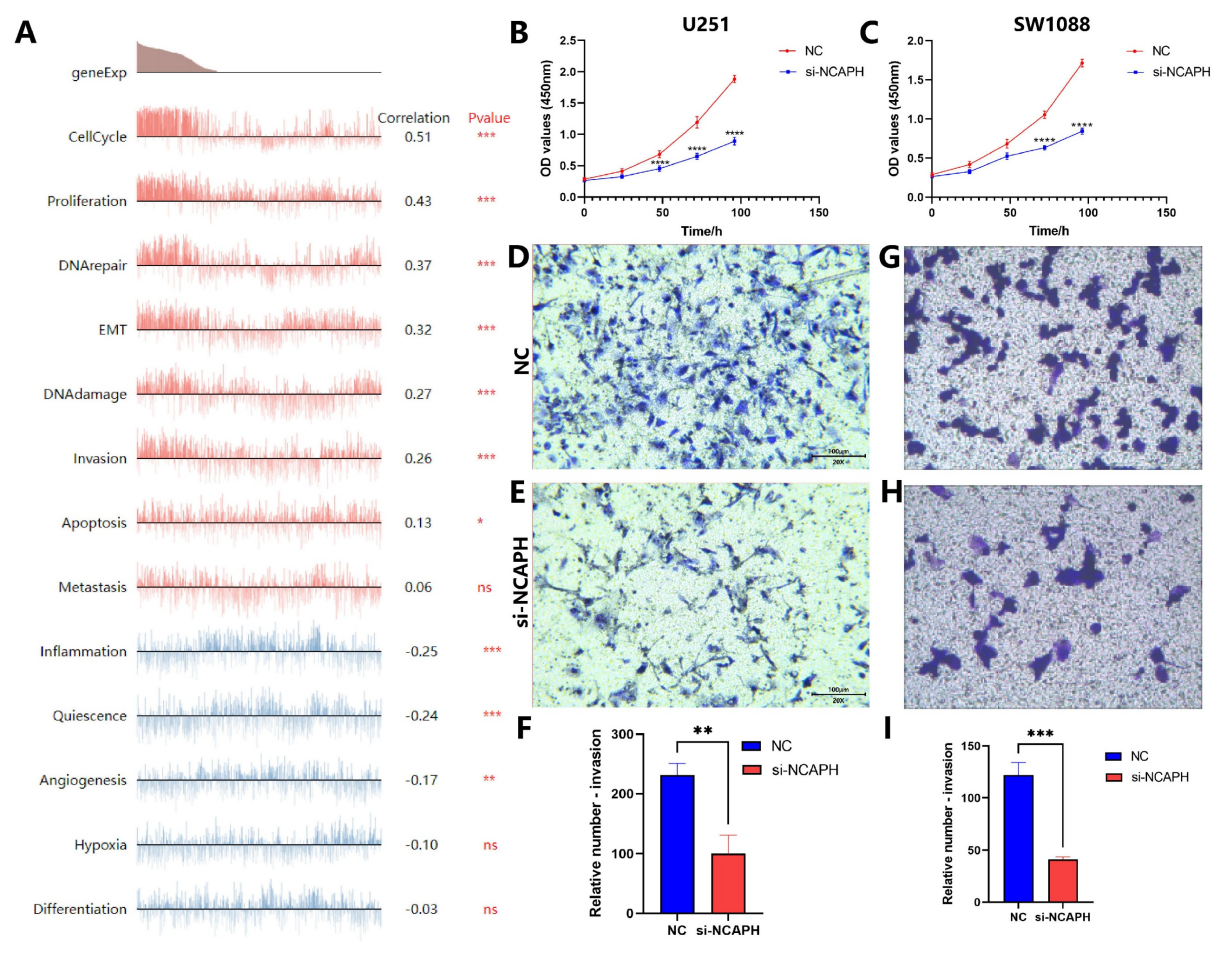
Analysis of association between NCAPH expression and biological functions. **A** Correlations between NCAPH and functional states in single-cell datasets.** B-C** CCK8 assay was performed on U251 and SW1088 cells to verify the effect of NCAPH on cell proliferation. **D-I** Transwell assay was performed on U251 and SW1088 cells to verify the effect of NCAPH on cell invasion.

To explore which biological functions of NCAPH are involved in gliomas, we entered the CancerSEA database 36, and data analysis based on Fibin MG Science 2018 (Brain) found that NCAPH was positively correlated with cell cycle, proliferation, DNA damage and repair, EMT, invasion, and apoptosis, while negatively associated with inflammation, quiescence and angiogenesis (Figure 11A). In order to further verify the above conclusions, we knocked down the mRNA expression of NCAPH in U251 and LGG cell line SW1088, respectively. CCK8 and Transwell experiments showed that after knocking down NCAPH, cell proliferation and invasion were reduced, which was consistent with the above results (Figure 11B-I). Interestingly, we found that high expression of NCAPH was negatively correlated with inflammation, which may be because high expression of NCAPH inhibited the protective immune response of the body, and the specific mechanism of its occurrence needs to be further studied.

## 4. Discussion

Two distinct subtypes of coagulin, known as coagulin I and coagulin II, comprise a protein complex consisting of structural maintenance parts SMC2 and SMC4, along with three non-SMC protein complex subunits: NCAPD2, NCAPG, and NCAPH 37, 38. Studies have shown that NCAPH may act as an oncogene in different tumor types. Elevated levels of NCAPH expression have been observed in tissues from patients with breast cancer 39, prostate cancer 40 and endometrial cancer 41 compared to normal tissues. Furthermore, patients with high NCAPH expression levels tend to exhibit a poorer prognosis. NCAPH can promote the proliferation, invasion and migration of tumor cells in many cancers 15, 42, and affect cell apoptosis 12, including glioblastoma 43. However, no researchers have explored the role of NCAPH in LGG in depth. Therefore, this study attempts to show the expression level of NCAPH in LGG, its impact on patient prognosis, potential biological function, correlation with immune cell infiltration and immunotherapy, and potential therapeutic agents.

The investigation in our study focused on the expression of NCAPH across a range of human tissues, encompassing brain tissues, brain cells, and pan-cancer samples. Our findings revealed elevated levels of NCAPH expression in normal liver, skin, esophagus, and other tissues, with comparatively lower expression observed in skeletal muscle and brain tissue. Furthermore, NCAPH expression was found to be highest in lymphoma, leukemia, and bone tumor cells, followed by brain tumor cells. Specifically, the mRNA expression levels of SF268, CNE-G_84, SF172, and GB-1 cells were highest in brain tumor cells. In pan-cancer analysis, NCAPH mRNA expression levels were found to be elevated across a range of tumor types, including LGG. This expression pattern is affected by gene mutation and chromosomal variation, and can cause poor prognosis in patients with multiple tumors, including LGG.

It is well known that genetic mutations and abnormal DNA methylation can cause up-regulation of many oncogenes, thus affecting the occurrence and development of tumors 44, 45. This study used gene expression profile data to reveal that NCAPH is overexpressed in almost all cancers, including LGG, which is consistent with the conclusions of previous investigators. This research focused on exploring the role of NCAPH in LGG, so we further studied the influence of NCAPH on the prognosis of LGG patients. Through multi-omics analysis and multilevel validation, we determined that NCAPH is an important target for poorer outcomes in patients with LGG.

Currently, study on NCAPH primarily centers on specific malignancies. Investigations have showed that high levels of NCAPH can stimulate the proliferation and dissemination of gastric cancer cells through the modulation of DNA damage response, thereby influencing the advancement of gastric cancer 46. Furthermore, heightened expression of NCAPH has been observed in bladder cancer, where it facilitates the proliferation of bladder cancer cells and suppresses their programmed cell death by activating the MEK/ERK pathway, consequently impacting the progression of bladder cancer. 13. Zhang et al. revealed that NCAPH can promote the proliferation, migration, invasion and epithelial-mesenchymal transformation (EMT) process of breast cancer cells by activating the PI3K/AKT signaling pathway 47. Other researches have also found that NCAPH can activate the AKT/mTOR pathway by up-regulating AURKB mRNA and protein expression, thereby promoting the proliferation, migration and invasion of breast cancer cells, and inhibiting the apoptosis of cisplatin-resistant breast cancer cells 48. Besides, NCAPH expression was increased in lung cancer 49, cervical carcinoma 42, colon cancer 12, serous ovarian cancer 50 and kidney cancer 51, all of which were involved in cancer progression. Nevertheless, the role of NCAPH in low-grade gliomas remains unclear. Our LGG enrichment analysis based on GSEA-GO/KEGG showed that metabolism-related pathways were mostly negatively enriched, such as dicarboxylic acid catabolic process, inhibitory synapse assembly, glutamate metabolic process and glutamate receptor signaling pathway, while regulation of cell cycle and DNA replication was positively enriched. Of course, we also found that NCAPH was significantly associated with glioma, non-small cell lung cancer, pancreatic cancer, colorectal cancer, and bladder cancer. The above enrichment analysis results suggest that we can explore the relationship between glioma and NCAPH in the biological processes such as amino acid metabolism, cell cycle, DNA replication, etc., so as to explore the potential pathogenic mechanism and find treatment methods in the future. Chen et al. found that NCAPH plays crucial roles in the proliferation and invasion of glioma cells. However, no relevant experimental verification has been conducted 52. In contrast, our experiments analyzed and verified both low-grade and high-grade gliomas, further confirming the role of NCAPH. Qin et al. found that the expression level of NCAPH was higher in female GBM. However, they did not further explore the impact of high NCAPH expression on the survival prognosis of patients 53. In contrast, we have verified the expression of NCAPH in different grades of gliomas, and our study is more comprehensive. In summary, our study has conducted a more thorough and detailed exploration of the expression of NCAPH in gliomas, especially in low-grade gliomas, as well as its impact on the prognosis of LGG patients, and the influence of NCAPH expression on tumor proliferation and invasion.

Nowadays, more and more people pay attention to tumor immunotherapy, and immunotherapy is gradually applied in clinical practice, but the overall clinical efficacy has not been significantly improved 54. Therefore, there is an urgent need to explore immune-based genes as tumor therapeutic targets. Prior research has demonstrated the impact of immune cells within the TME on tumor progression, highlighting their significance in immunotherapy 55, 56. Numerous studies have identified cytotoxic CD8+T lymphocytes (CTL), CD4+T helper cells 1 (Th1 cells), and natural killer cells (NK) as primarily exerting anti-cancer effects, while CD4+Th2 cells and myeloid-derived suppressor cells (MDSCs) are associated with pro-cancer effects 57. Our study delved into the relevance between NCAPH expression and immune cell infiltration in LGG, revealing a positive relationship between NCAPH levels and Tgd cells, MEP, endothelial cells, erythrocytes, fibroblasts, Macrophages-M0 and Tregs cells, while a negative associated with NK cells, Gamma Delta T cells, Platelets, class-switched memory B cells, hepatocytes, neurons, monocytes and neutrophils. Our results suggest that NCAPH may form an inhibitory immune microenvironment by promoting fibroblasts, Treg cells and inhibiting NK cells, class-switched memory B cells, Gamma Delta T cells under the regulation of chemokines and cytokines.

Our study has several limitations. First, the lack of clinical sample validation necessitates caution in interpreting NCAPH's clinical applicability. Second, the mechanisms by which NCAPH modulates immune tolerance (e.g., via PD-L1 or Treg recruitment) remain speculative. Future studies with spatially resolved transcriptomics or co-culture models are warranted to dissect these interactions.

At present, there are few studies on NCAPH in low-grade gliomas, and few studies on tumor immunotherapy and the selection of immunotherapy targets. Chen et al. discovered that NCAPH has the ability to upregulate the expression of PD-L1 by impeding the degradation of β-catenin protein in clear cell renal cancer cells, causing the enhancement of aerobic glycolysis and immune tolerance in these cells. This suggests that NCAPH could potentially serve as a therapeutic target for reversing immune tolerance in patients with clear cell renal cancer 51. Building upon this finding, we administered anti-PD-L1, anti-PD-1, and anti-PD-L1/PD-1 treatments to patients with LGG. Our findings indicated that LGG patients who exhibited a positive response to anti-PD-L1 treatment displayed elevated levels of NCAPH expression, and their AUC value reached 0.656, so it had certain predictive significance. Nevertheless, there was no statistically significant disparity in OS rates among patients with low and high NCAPH expression levels in LGG. Consequently, further substantiation is required to establish PD-L1 as a viable therapeutic target for LGG patients exhibiting elevated NCAPH expression. Furthermore, our drug sensitivity analysis showed that LGG patients with high NCAPH expression were particularly responsive to temozolomide, a chemotherapy drug commonly used in glioma treatment. This finding implies the potential for combining temozolomide with existing treatments for patients with high NCAPH expression in future clinical practice.

## 5. Conclusion

Our research demonstrates a significant upregulation of NCAPH in LGG, with a strong correlation to poor prognosis. NCAPH's involvement in coordinating immune cell infiltration into the tumor microenvironment suggests a role in promoting immune tolerance in LGG, making it a potential target for immunotherapy. The sensitivity of LGG patients with high expression of NCAPH to chemotherapeutic agents such as temozolomide also suggests the potential therapeutic effect of chemotherapy combined with immunotherapy. Ultimately, NCAPH emerges as a plausible prognostic biomarker in LGG patients, holding promise for the advancement of drug development and immunotherapeutic strategies.

## Figures and Tables

**Table 1:** Univariable and multivariable Cox regression analysis of the risk factors for LGG patients.

Subject characteristics		Univariable OR (95% CI)	P-value	Multivariable OR (95% CI)	P-value
WHO grade	G2	1 (Reference)		Reference	
	G3	3.023	6.96e-08	1.668	0.0452
IDH status	WT	1 (Reference)		Reference	
	Mut	0.184	1.01e-20	0.371	0.0002
1p/19q codeletion	Non-codel	1 (Reference)		Reference	
	Codel	0.401	6.99e-05	0.845	0.6130
Primary therapy outcome	PD	1 (Reference)		Reference	
	SD	0.439	8.1e-05	0.379	0.0003
	PR	0.172	3.55e-05	0.179	0.0013
	CR	0.119	9.56e-08	0.151	5.41e-06
Gender	Female	1 (Reference)		Reference	
	Male	1.112	0.5417	1.766	0.0114
Age	<= 40	1 (Reference)		Reference	
	> 40	2.898	9.65e-09	3.079	1.7e-06
Histological type	Astrocytoma	1 (Reference)		Reference	
	Oligoastrocytoma	0.649	0.0594	1.569	0.0960
	Oligodendroglioma	0.574	0.0047	0.605	0.0837
NCAPH	Low	1 (Reference)		Reference	
	High	2.648	6.13e-07	1.459	0.0142

WT, wide type; Mut, mutant; PD, progressive disease; SD, stable disease; PR, partial response; CR, complete response
